# Photothermal and adsorption effects of silver selenide nanoparticles modified by different surfactants in nursing care of cancer patients

**DOI:** 10.1080/14686996.2020.1800367

**Published:** 2020-09-01

**Authors:** Xingju Yang, Chen Wang, Xiaoxu Zhang, Yue Wang, Feng Gao, Lixia Sun, Wenli Xu, Changxiu Qiao, Guiqin Zhang

**Affiliations:** a Department of Nursing, Jinan People’s Hospital Affiliated to Shandong First Medical University, Jinan China; b Neurointerventional Department, The First Affiliated Hospital of Zhengzhou University, Zhengzhou, China; c The Intensive Care Unit (ICU), Jinan People’s Hospital Affiliated to Shandong First Medical University, Jinan China; d Tumor Department, Jinan People’s Hospital Affiliated to Shandong First Medical University, Jinan China; e Nursing School of Binzhou Medical University, Binzhou, China

**Keywords:** Synthesis, characterization, photothermal, silver selenide, surfactants, 212 Surface and interfaces, 503 TEM, STEM, SEM, 301 Chemical syntheses / processing

## Abstract

Silver selenide nanoparticles have advantages of low cytotoxicity, desirable near-infrared light response characteristics, and easy surface modification, which attract increasing attention in chemo-photothermal therapy and nursing care of cancer patients. In this contribution, we synthesized Ag_2_Se nanoparticles modified by the surfactant of cetyltrimethylammonium bromide (CTAB) using a ligand exchange strategy. Their microstructure and composition were characterized by transmission electron microscopy, X-ray diffraction, X-ray Photo-electronic Spectroscopy, and Fourier transform infrared spectroscopy. The CTAB modified Ag_2_Se nanoparticles exhibited a uniform diameter distribution centered at ~12 nm. In order to investigate the photothermal and adsorption effects of CTAB-Ag_2_Se nanocomposites, we also prepared sodium dodecyl benzene sulfonate (SDBS) modified Ag_2_Se nanoparticles to make a comparison. The CTAB-Ag_2_Se nanoparticles showed high photothermal properties, a photothermal conversion efficiency of 20.1% and a high drug adsorption performance of 48.2 μg/mg. Importantly, the CTAB-Ag_2_Se-DOX presented an MCF-7 cell activity of only 27.3% under near-infrared radiation. The results revealed that the surface-modified Ag_2_Se nanoparticles with CTAB had stronger antitumor ability.

## Graphical Abstract

**Figure:**
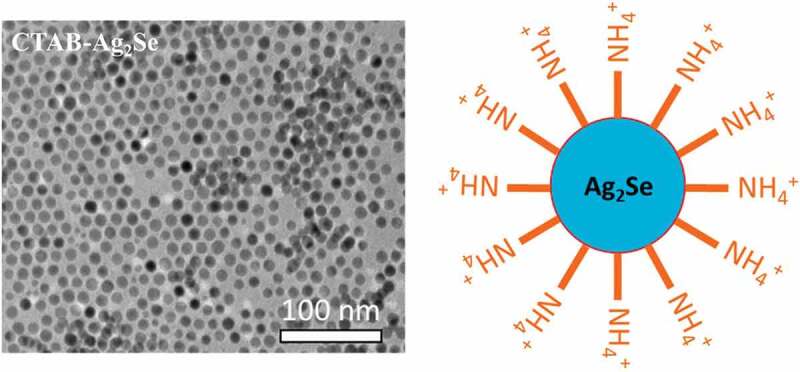

## 1. Introduction

In recent years, photothermal therapy (PTT) has received widespread attention as an emerging therapy in cancer treatment, which uses the photothermal properties of photosensitive materials to trigger tumor cell ablation [1,2]. Nanomaterials for PTT are divided into two categories: organic and inorganic. Traditional organic compounds are indocyanine green, polyaniline, etc. [3–5]. Their disadvantages are low light-to-heat conversion rate and severe photobleaching. Inorganic photothermal materials are based on noble metal nanoparticles [6–8]. The high price of noble metals stimulates the search for cheaper alternatives.

Metal selenides have recently attracted increasing attention because of their excellent optical properties, easiness in surface-functionalization and stability; therefore, they have been widely used in photocatalysis, optoelectronics, and energy storage [9–13]. Particularly, silver selenide possesses good biocompatibility and high efficiency in converting light to heat, making it a promising material in photothermal therapy of cancer patients [14–16]. However, there are several issues that need to be concerned for cancer treatment based on PTT. Firstly, the insufficient light penetration depth may lead to incomplete elimination of cancer cells, which is the main hidden danger of thorough cancer treatment [17,18]. Secondly, the dispersibility of nanomaterials is also a problem to be solved [1,19]. Last but not least, the shortcomings of monotherapy cannot be ignored, current antitumor research tends to favor multiple approaches [20,21]. The combination of photothermal and chemotherapy can solve the hidden danger of recurrence in single PTT, and it is necessary to use photosensitive materials to explore more comprehensive treatment methods [22]. Recently, Li et al. prepared the Ag_2_Se quantum dots by the introduction of bovine serum albumin (BSA) as dispersant [23]. The Ag_2_Se@BSA composite showed good dispersibility, high biocompatibility and remarkable photothermal conversion. However, the drug delivery and photothermal therapy of Ag_2_Se@BSA need to for further combine with the extra mesoporous silica nanoparticles.

In this study, we reported the facile synthesis of Ag_2_Se nanoparticles modified by different surfactants including cetyltrimethylammonium bromide (CTAB), and sodium dodecyl benzene sulfonate (SDBS). Importantly, the CTAB-Ag_2_Se composite demonstrated improved antitumor ability. The CTAB as a surfactant can not only enhance the dispersive ability and biocompatibility of Ag_2_Se nanomaterials but also provide a porous layer on the Ag_2_Se surface to achieve higher drug loading capacity. At the same time, due to the excellent photothermal performance of Ag_2_Se nanoparticles, this strategy can achieve the expected purpose.

## 2. Experimental section

### 2.1 Materials

All chemical reagents were used without further purification. Dodecanethiol (98%), CTAB, SDBS, concentrated HCl (37%), ethanol, doxorubicinhydrochloride (DOX·HCl), and dimethyl sulfoxide (DMSO) were purchased from Aladdin Chemical Inc. (Shanghai, China). Michigan cancer foundation-7 (MCF-7) cells were obtained from the Jinan City People’s Hospital. The 3-(4,5-dimethyl-2-thiazolyl)-2-5-diphenyl-2 H-tetrazolium-bromide (MTT) was purchased from Shanghai Pumai Biotechnology.

### 2.2 Synthesis of CTAB and SDBS modified Ag_2_Se nanoparticles

The oil-soluble dodecanethiol modified Ag_2_Se nanoparticles were synthesized according to a reported study [24]. CTAB and SDBS modified Ag_2_Se nanoparticles were prepared using the ligand exchange strategy on the basis of above dodecanethiol-Ag_2_Se nanoparticles. Detailedly, 100 mg dodecanethiol-Ag_2_Se precursors were dissolved in 20 ml chloroform and 20 ml H_2_O, followed by addition with 4 g CTAB. The mixtures were kept at 50°C under the N_2_ protection and stirred for 4 h. After cooling down to room temperature, the products were collected and washed with ethanol and re-dispersing in distilled water. Similarly, the SDBS-Ag_2_Se nanoparticles were obtained by replacing dodecanethiol ligand with SDBS. Briefly, 100 mg dodecanethiol-Ag_2_Se precursors and 4 g SDBS were dispersed in 40 ml chloroform, which was then refluxed at 60°C under the N_2_ protection and stirred for 12 h. After the reaction, the collected samples were washed with chloroform and ethanol and re-dispersing in distilled water.

### 2.3 Characterization

The morphology of the prepared samples was characterized by transmission electron microscopy (TEM; JEM 2100, Jeol, Tokyo, Japan), and elemental mapping images were obtained using energy dispersive spectroscopy (EDS). The phase compositions of the samples were determined by X-ray diffraction (XRD; D/max-2500, Cu Kα). Fourier transform infrared (FTIR) spectra were recorded with a model-8400s FTIR spectrometer (Shimadzu, Japan). N_2_ adsorption-desorption isotherms were obtained using a Builder SSA-4200 instrument at 77 K. X-ray photoelectron spectroscopy (XPS) spectra were recorded by a PHI5600X of PerkinElmer from USA. A 980 nm diode laser was obtained from Changchun New Industries Optoelectronics Technology Co. Ltd. (Jilin, China).

### 2.4 Measurement of the photothermal effect

To measure the photothermal performance, sample nanoparticles were dispersed in deionized water with different concentrations (0, 0.0625, 0.125, 0.25, 0.5 and 1 mg/mL). To evaluate the photothermal performance, 2 mL of deionized water, CTAB-Ag_2_Se and SDBS-Ag_2_Se aqueous dispersions with different concentrations were irradiated for 10 min by an external adjustable power (0–1.66 W/cm^2^) 980 nm semiconductor laser device. The output power was independently calibrated using an optical power meter. The temperature evolution of the aqueous dispersions under irradiation was recorded by using a hand-held thermometer every 2 min. To measure the photothermal conversion efficiency (*η*), the temperature change of the CTAB-Ag_2_Se and SDBS-Ag_2_Se dispersions were recorded as a function of time under continuous irradiation of the 980 nm laser with a power density of 1.66 W/cm^2^ until the solution reached a steady-state temperature. The *η* value was calculated by eq (1) [25]:

(1) $$\eta =\frac{hS\left(T_{m}-T_{s}\right)-Q_{s}}{I\left(1-10^{-A_{\lambda }}\right)}$$

where *h* is the heat transfer coefficient, *S* is the surface area of the container, *T_m_* is the equilibrium temperature, *T_s_* is the ambient temperature of the surroundings, *Q_s_* stands for the heat input due to the absorption of water, *I* is the incident laser power, and *A_λ_* is the absorbance of the cesium tungsten bronze nanomaterial at 980 nm [26].

### 2.5 Loading and release of DOX

Firstly, 1.0 mg of CTAB-Ag_2_Se and SDBS-Ag_2_Se nanoparticles were dispersed into DOX phosphate-buffered saline (PBS) solution (5.0 mL, 1 mg/mL). Next, the DOX-loaded samples were centrifuged at 8000 rpm and then washed repeatedly with PBS solution to remove free DOX molecules. All the washing solutions were collected and measured by a UV-vis spectrophotometer. The measurements were performed in triplicate, and the loading amount of DOX was calculated using eq. (2).

(2) $$Drug\;loading\;capacity=\frac{M_{DOX}}{M_{NPs}+M_{DOX}}$$

where M_DOX_ is the mass of DOX, M_NPs_ is the mass of CTAB-Ag_2_Se nanoparticles. The unit of drug loading capacity is μg/mg. The release behavior of DOX was studied in PBS solution (pH 7.4 and pH 5.0) at room temperature. DOX-loaded CTAB-Ag_2_Se and SDBS-Ag_2_Se nanocomposites were dispersed in 30 mL of PBS solution and stirred. The supernatants were centrifugated every several hours to measure the released DOX and then replaced with fresh PBS solution. All the tests were carried out in triplicate and the average values are reported.

### 2.6 In vitro *cell toxicity assay*

The cytotoxicity of CTAB-Ag_2_Se nanocomposites was evaluated by a standard MTT assay using MCF-7 cells as models. MCF-7 cells were inoculated in 96-well plate at 104/well for 6 groups. Five parallels for each group were conducted. CTAB-Ag_2_Se suspensions (0, 0.0625, 0.125, 0.25, 0.5, and 1 mg/mL) were added to the cells. After incubating for 24 h, the medium was then aspirated using a pipette and fresh medium was added to the culture dishes. Next, the cancer cells were irradiated with a 980 nm near-infrared (NIR) laser for 5 minutes, washed gently with PBS three times to wash away the nanomaterials that were not phagocytized by the cells, then placed in an incubator for further 4 hours. Subsequently, 20 μL MTT solutions (5 mg/mL) were injected and maintained for 4 h after incubation. Then, the medium was removed and 150 μL DMSO was injected. After shaking the plate for 15 min, the absorbance was measured at 490 nm to calculate the cell survival rate.

## 3. Results and discussion

**Figure 1.**
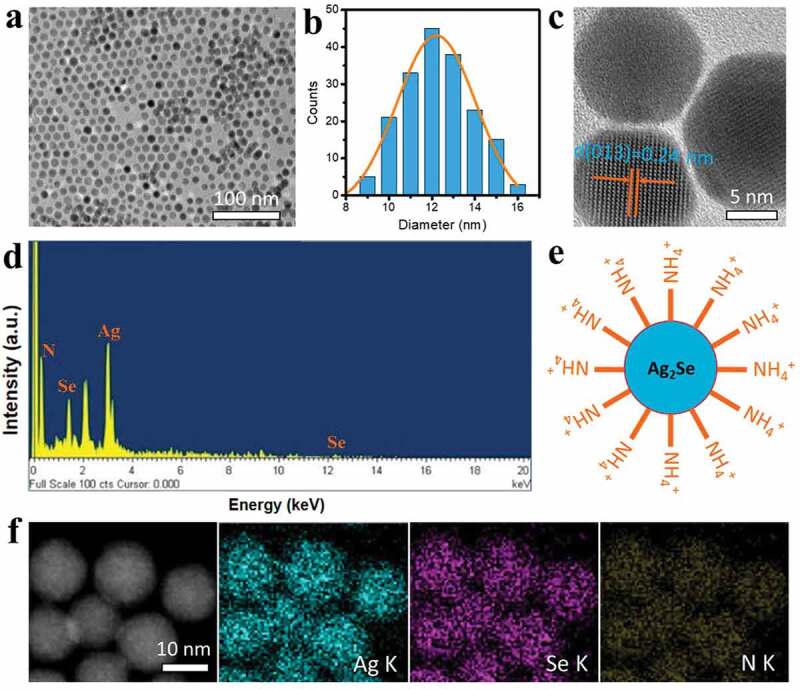
TEM image (a), diameter distribution (b), high-resolution TEM image (c), and EDS spectrum (d) of CTAB-Ag_2_Se nanoparticles. (e) Illustration of CTAB-Ag_2_Se nanoparticles with exposed NH_4_^+^ group. (f) elemental maps of CTAB-Ag_2_Se nanoparticles.

The morphology of the as-prepared CTAB- Ag_2_Se nanoparticles has been studied by TEM technique, as shown in Figure 1(a). These particles possess excellent dispersion and uniform distribution in a diameter of around 12 nm (Figure 1(b)). The amine salt type cationic surfactant of CTAB is able to effectively restrain the intergranular agglomeration through the surface modification for Ag_2_Se nanoparticles. Figure 1(c) displays the high-resolution TEM image of CTAB-Ag_2_Se nanoparticles, we can observe a group of well-resolved lattice fringe with the interplanar distance of 0.24 nm, it is ascribed to the (0 1 3) plane of the orthorhombic phase of Ag_2_Se [27]. The EDX spectrum in Figure 1(d) presents the signals from element N, Ag, and Se, indicative of the successful preparation of CTAB-Ag_2_Se nanocomposites. The peak at 2.2 keV is the Au signal, which has been used to improve the conductivity of the sample. The schematic diagram of such a composite nanoparticle is depicted in Figure 1(e) where Ag_2_Se nanoparticle is covered by a layer of CTAB with the exposed NH_4_^+^ group. The structural information of CTAB-Ag_2_Se nanoparticles can be further obtained from elemental mapping results in Figure 1(f). The Ag, Se, and N elements demonstrate the homogeneous distributions over the nanoparticles, confirming the above results. For comparison, the anionic surfactant SDBS modified Ag_2_Se nanoparticles have been also synthesized, and Fig. S1 gives their TEM image and schematic diagram.

**Figure 2.**
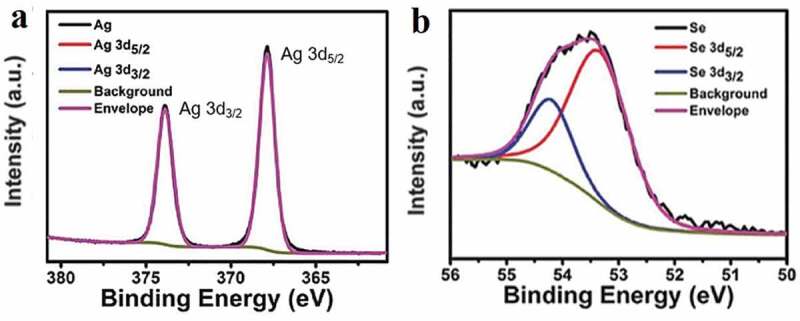
XPS spectra of the as-prepared Ag_2_Se nanoparticles around the Ag 3d (a) and Se 3d (b) edges.

XPS analysis has been employed to study the chemical composition and oxidation states of prepared material. As shown in Figure 2(a), the higher binding energies (compared to Ag(0)) located at 367.9 eV and 374.2 eV stand for Ag 3d_5/2_ and Ag 3d_3/2_, respectively, indicating the presence of oxidized Ag(l) species in Ag_2_Se [28]. The band situated at 54.2 eV in Figure 2(b) is assigned to Se 3d containing two contributions from Se 3d_5/2_ and Se 3d_3/2_, which agrees well with the reported result of Ag_2_Se nanomaterial [29].

**Figure 3.**
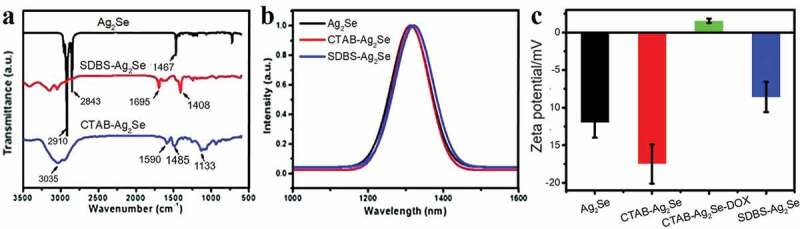
FTIR transmittance (a), spectra are vertically offset for clarity, photoluminescence emission spectra (b), and zeta potentials (c) of Ag_2_Se, CTAB-Ag_2_Se, and SDBS-Ag_2_Se nanoparticles.

The FTIR spectra in Figure 3(a) confirm the surface modification of Ag_2_Se nanoparticles by CTAB and SDBS surfactants. The three obvious peaks at 2910 cm^−1^, 2843 cm^−1^, and 1467 cm^−1^ are characteristic signals of 1-dodecanethiol. Compared to the pure Ag_2_Se, the distinctive peaks at 1407 cm^−1^ and 1693 cm^−1^ of SDBS-Ag_2_Se sample correspond to the symmetric and asymmetric COO^−^ stretching modes [30], indicating that SDBS exists on the surface of Ag_2_Se nanoparticles. In the spectrum of CTAB-Ag_2_Se sample, we can observe several characteristic peaks located at 3035 cm^−1^, 1484 cm^−1^, and 1589 cm^−1^, they are ascribed to the NH_4_^+^ stretching vibration, NH_4_^+^ deformation vibration, and the N–H bending vibration, respectively [31]. Both CTAB-Ag_2_Se and SDBS-Ag_2_Se nanocomposites retain the NIR II fluorescence properties of Ag_2_Se nanoparticles (Figure 3(b)), their emission wavelengths are around 1320 nm. The zeta potentials of Ag_2_Se, CTAB-Ag_2_Se, and SDBS-Ag_2_Se nanoparticles have been recorded and provided in Figure 3(c). The zeta potential of CTAB-Ag_2_Se was about −12.13 mV, while it changed to 1.68 mV when DOX was installed. The change in charge of zeta potential further illustrated the load of DOX. However, the zeta potential of the SDBS-Ag_2_Se was only −8.04 mV, which indicated that the CTAB-Ag_2_Se had a better adsorption amount of DOX than that of SDBS-Ag_2_Se.

**Figure 4.**
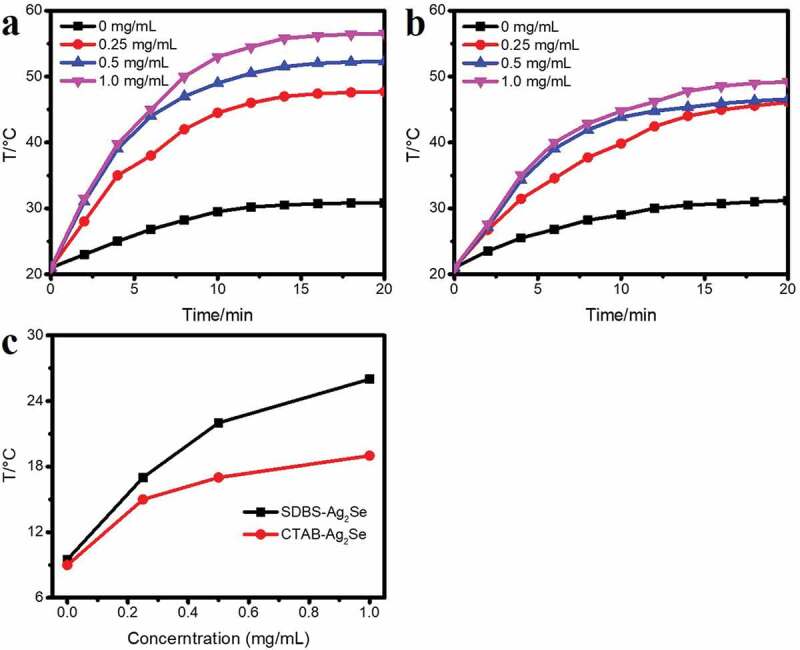
NIR-induced heat generation in SDBS-Ag_2_Se (a) and CTAB-Ag_2_Se (b) suspensions at different concentrations. (c) NIR-induced temperature increase for SDBS-Ag_2_Se and CTAB-Ag_2_Se at different concentrations.

The photothermal performance of the samples is shown in Figure 4. The samples of CTAB-Ag_2_Se and SDBS-Ag_2_Se nanocomposites were dispersed in distilled water at different concentrations (0, 0.25, 0.5, and 1 mg/mL) to study the photothermal heating effect. Subsequently, they were irradiated by NIR 980 nm laser irradiation at a power density of 1.66 W cm^−2^ for 20 min. As demonstrated in Figure 4(a, b), the temperature only slightly increased in distilled water, while obvious increases were observed in CTAB-Ag_2_Se and SDBS-Ag_2_Se. SDBS-Ag_2_Se is more responsive to temperature and the temperature can be controlled from 20.6°C to 56.8°C by adjusting the composite content. To further investigate the photothermal transduction ability (Figs. S2a, b), we evaluated the temperature changes of CTAB-Ag_2_Se and SDBS-Ag_2_Se suspension (1 mg/mL) under different laser power densities of 980 nm continuous-wave lasers. The photothermal curve shows that the CTAB-Ag_2_Se and SDBS-Ag_2_Se also have the strong laser power-dependent photothermal effects. These results indicated that SDBS-Ag_2_Se nanocomposites could convert the energy of a 980 nm laser into heat more quickly and efficiently. Thermal properties are also very important for achieving high photothermal conversion. As can be seen intuitively from Figure 4(c) and Fig. S2c, obvious rapid temperature changes can be observed in the groups of SDBS-Ag_2_Se (ΔT = 37°C) and CTAB-Ag_2_Se (ΔT = 27°C) with the concentration of 1 mg/mL and a power density of 1.66 W/cm^2^. The results indicate that the Ag_2_Se nanoparticles modified by surfactants have concentration-dependent temperature elevations and excellent photothermal conversion. Therefore, we believe that SDBS-Ag_2_Se nanocomposites have the superior potential for photothermal therapy.

**Figure 5.**
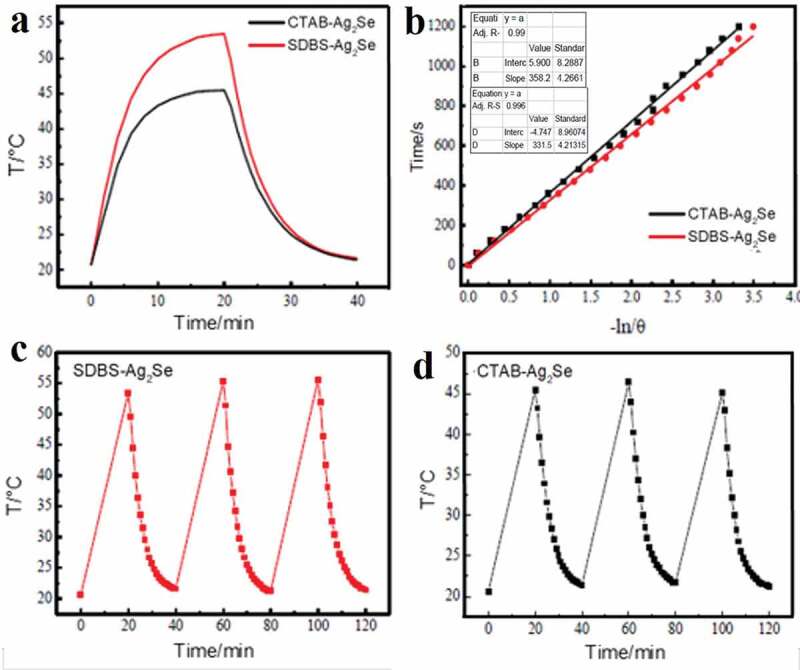
(a) The photothermal response of SDBS-Ag_2_Se aqueous dispersion (2 mL, 0.5 mg/mL) under a pulse of laser irradiation (1.66 W, 980 nm). (b) Plots of cooling time (after 20 min) versus negative natural logarithm of the driving force temperature (-ln/θ) obtained from cooling stage as shown in (a). SDBS-Ag_2_Se (c) and CTAB-Ag_2_Se (d) aqueous dispersion photothermal cycle diagram.

In order to further study its photothermal conversion efficiency, according to Figure 5(a, b), the photothermal conversion ability of the material has been evaluated by the photothermal conversion efficiency (η). The dispersion liquid (2 ml, 0.5 mg/ml) has been irradiated with a 980 nm laser for 20 minutes, and then naturally cooled. Figure 5(b) shows the curve of cooling time t-(-lnθ), calculated according to the method in the literature [32], the photothermal conversion efficiencies of SDBS-Ag_2_Se and CTAB-Ag_2_Se are 22.9% and 20.1%, respectively. Anionic surfactant SDBS may have a larger influence on the electronic structure of the Ag_2_Se nanoparticles, thus leading to the superior photothermal property. As shown in Figure 5(c, d), the temperature changes of SDBS-Ag_2_Se and CTAB-Ag_2_Se dispersion liquid (0.5 mg/ml) have no obvious reduction after repeatedly turning on or off the laser for 3 times, implying that the colloidal SDBS-Ag_2_Se and CTAB-Ag_2_Se nanocomposites had excellent photothermal stability.

**Figure 6.**
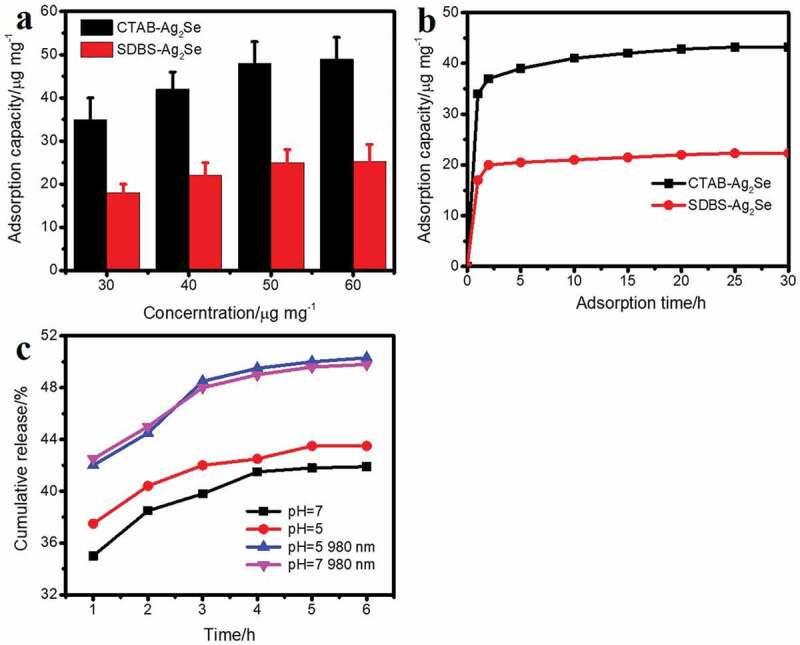
(a) Adsorption capacity of CTAB-Ag_2_Se and SDBS-Ag_2_Se nanoparticles at different concentrations. (b) The adsorption amount of the sample at the maximum concentration with time. (c) Drug release profiles of CTAB-Ag_2_Se in pH 7 and pH 5.0 PBS within 6 h.

The SDBS-Ag_2_Se and CTAB-Ag_2_Se nanocomposites are promising candidates to use as drug delivery platforms for PTT based on their structural characteristics. Thus, the anticancer drug DOX has been used to evaluate the drug adsorption behaviors of both nanomaterial carriers. At the same concentration, the drug adsorption capacity of CTAB-Ag_2_Se is significantly higher than that of SDBS-Ag_2_Se. As shown in Figure 6(a), the drug loading capacity of CTAB-Ag_2_Se is 48.2 μg/mL while it is only 25.3 μg/mL for SDBS-Ag_2_Se with the concentration of 50 μg/mg. Figure 6(b) displays the adsorption capacity of SDBS-Ag_2_Se and CTAB-Ag_2_Se at the maximum concentration as the loading time changes, and the adsorption amount of the sample gradually increases and tends to the absorption equilibrium. It is clear that CTAB-Ag_2_Se has the excellent adsorption ability. The reason may be due to its porous structure (larger pore diameter of 0.9–1.1 nm for CTAB-Ag_2_Se than 0.7–0.8 nm for SDBS-Ag_2_Se, as shown in Fig. S3) which was formed by treatment the sample with a certain ratio of methanol-hydrochloric acid reflux. The drug release behavior of CTAB-Ag_2_Se-DOX has been used to investigate the efficacy of chemotherapy in cancer treatment. For this, pH 7.4 PBS with or without near-infrared light and pH 5.0 PBS are used to mimic normal tissues and the microenvironments of tumor tissues with or without NIR light, respectively. Figure 6(c) shows the cumulative DOX release from the CTAB-Ag_2_Se-DOX under different conditions. The percentage cumulative drug release of CTAB-Ag_2_Se could reach up to 44.63% under pH 5.0, which was higher than that under pH 7.4. The release of this drug may be due to the weakening of the electrostatic interaction between DOX and CTAB-Ag_2_Se under acidic conditions, which makes DOX easier to dissociate from CTAB-Ag_2_Se nanoparticles. Moreover, it is clearly seen that the behavior of DOX releasing has been enhanced under the irradiation, and the cumulative drug release in pH 5 and 7 are 49.16% and 48.31%, respectively. The drug release efficiencies are comparable to the reported results, such as mesoporous silica nanoparticles, nanoporous gold wires, and bovine serum albumin modified Ag_2_Se quantum dots [23,33,34]. It can be attributed to the fact that the Ag_2_Se nanomaterial effectively converts the infrared laser energy into heat, which weakens the interaction force between the drug and the nanomaterial, and thus helps the drug release.

**Figure 7.**
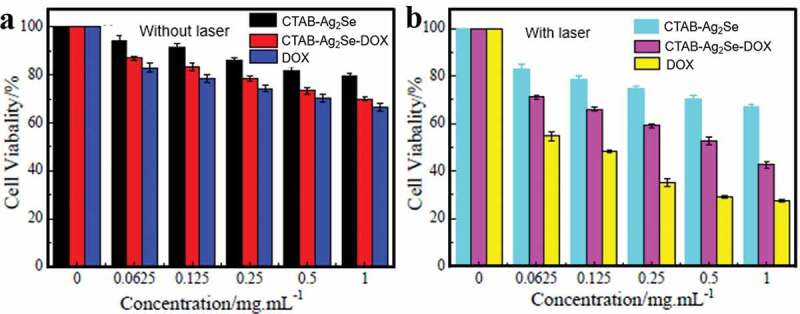
Viability of MCF-7 cells incubated with the control group and different concentrations of DOX, CTAB-Ag_2_Se, CTAB-Ag_2_Se-DOX without (a) and with (b) irradiation.

The CTAB-Ag_2_Se carrier has been chosen to evaluate the cytotoxicity towards MCF-7 cells based on its prominent photothermal effect and high drug adsorption efficiency. According to Figure 7(a), the viabilities of MCF-7 cells incubated with blank CTAB-Ag_2_Se nanoparticles at a high concentration of 1 mg/mL were above 80%, suggesting the high safety in the absence of NIR. Free DOX showed concentration-dependent toxicity to MCF-7 cells. In addition, when the concentration range is 0–1 mg/mL without NIR laser, CTAB-Ag_2_Se has been used as a drug carrier, and the cell activity decreased significantly with increasing concentration, from 83% to 66%, indicating that the anti-tumor efficiency of DOX released by nanocomposites was comparable to that of free DOX. To evaluate the effect, cells were irradiated with 980 nm laser, CTAB-Ag_2_Se and CTAB-Ag_2_Se-DOX were subjected to NIR laser at a power density of 1.66 W/cm^2^ under specific conditions. As shown in Figure 7(b), in the near-infrared irradiation group, the cell viability in the range of 0–1 mg/mL DOX is much lower than that of the free DOX without light, suggesting that the near-infrared laser irradiation has a significant effect on MCF-7 cell viability. However, for CTAB-Ag_2_Se with NIR radiation, an increase in the cytotoxicity of MCF-7 cells has been observed with increasing concentration of CTAB-Ag_2_Se, confirming that CTAB-Ag_2_Se can effectively absorb near-infrared light and convert it into thermal energy to induce MCF-7 cell apoptosis. For CTAB-Ag_2_Se-DOX with near-infrared radiation, more pronounced cytotoxicity is produced, and the cell activity is only 27.3% when the concentration of CTAB-Ag_2_Se-DOX is 1 mg/ml.

## 4. Conclusions

In summary, we have synthesized CTAB modified Ag_2_Se nanoparticles with low toxicity and improved antitumor ability by a ligand exchange method. TEM results show that the prepared CTAB-Ag_2_Se nanoparticles have a uniform diameter distribution centered at 12 nm. The CTAB layer enhances the dispersive ability and biocompatibility of Ag_2_Se nanomaterials, and also improves the drug loading capacity. The CTAB-Ag_2_Se nanocomposite exhibits a drug adsorption capacity of 48.2 μg/mg, which is much higher than that of SDBS-Ag_2_Se (25.3 μg/mg). Cytotoxicity tests demonstrated that CTAB-Ag_2_Se with high drug load had a stronger therapeutic effect. Hence, suitable surface modifiers have potential application value for photothermal nanomaterials in combination therapy, providing additional possibilities for future research on clinical therapeutic materials.

## Supplementary Material

Supplemental Material
