# Supplementary material for: Photothermal and adsorption effects of silver selenide nanoparticles modified by different surfactants in nursing care of cancer patients
Source: Sci Technol Adv Mater. 2020 Sep 1;21(1):584–92. doi: 10.1080/14686996.2020.1800367 (PMC7476516; doi:10.1080/14686996.2020.1800367)
Supplement: Supplemental Material [file TSTA_A_1800367_SM7215.pdf]

Supporting Information

Photothermal and adsorption effects of silver selenide nanoparticles modified by different surfactants in nursing care of cancer patients

Xingju Yang[1,†], Chen Wang[2,†], Xiaoxu Zhang[4], Yue Wang[4], Feng Gao[5], Lixia Sun[1], Wenli Xu[4], Changxiu Qiao[3,*], Guiqin Zhang[1,*]

[1] Department of nursing, Jinan people's Hospital Affiliated to Shandong First Medical University, Jinan 271199, China. Email: guiqinzhang12@163.com

[2] Neurointerventional Department, the First Affiliated Hospital of Zhengzhou University, Zhengzhou, 450052, China.

[3] Nursing School of Binzhou Medical University, Binzhou, 256603, China. Email: cxqiao2662@163.com

[4] The Intensive Care Unit (ICU), Jinan people's Hospital Affiliated to Shandong First Medical University, Jinan 271199, China

[5] Tumor Department, Jinan people's Hospital Affiliated to Shandong First Medical University, Jinan 271199, China

X. Yang[†] and C. Wang[†] equally contributed to this work.

Information Classification: General

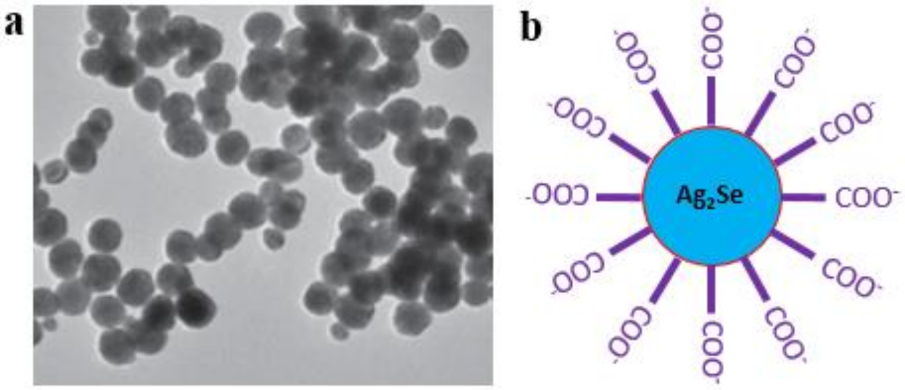

Fig. S1. TEM image (a) and Schematic diagram (b) of SDBS-Ag2Se nanoparticles.

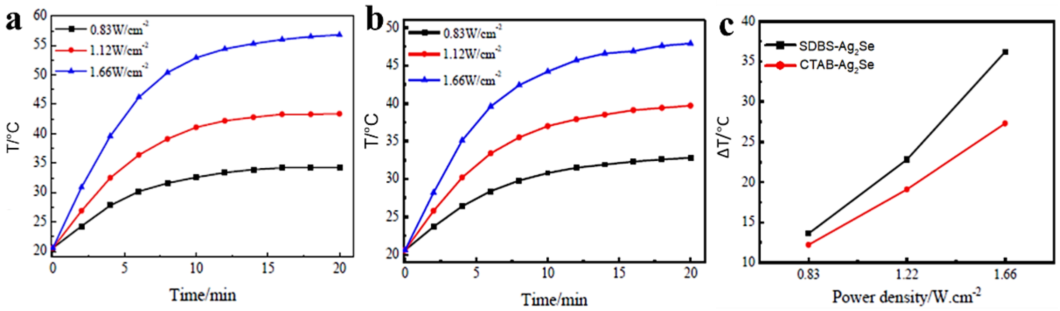

Fig. S2. NIR-induced heat generation of SDBS-Ag2Se (a) and CTAB-Ag2Se (b) suspensions irradiated with different power densities. (c) NIR-induced temperature difference of SDBS-Ag2Se and CTAB-Ag2Se irradiated with different power densities.

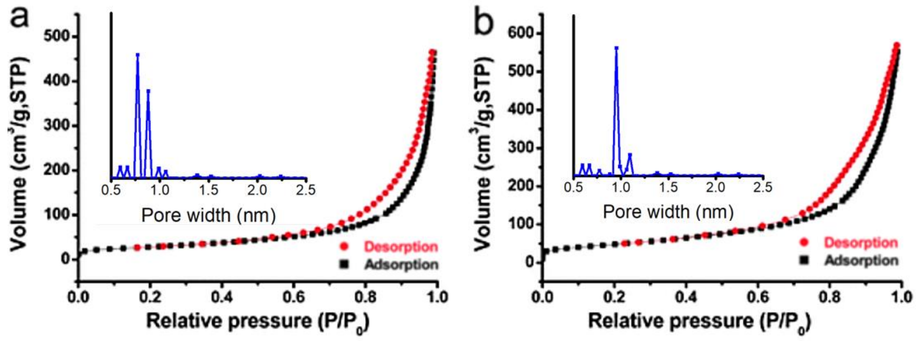

Fig. S3. N_2 adsorption-desorption isotherms of SDBS-Ag2Se (a) and CTAB-Ag2Se (b).

Information Classification: General
